# An assessment of randomized controlled trials (RCTs) for non-communicable diseases (NCDs): more and higher quality research is required in less developed countries

**DOI:** 10.1038/srep13221

**Published:** 2015-08-14

**Authors:** Hong Fan, Fujian Song

**Affiliations:** 1 Department of Social Medicine and Health Education, School of Public Health, Nanjing Medical University, Nanjing, P.R. China; 2 Norwich Medical School, University of East Anglia, Norwich, NR4 7TJ, U.K

## Abstract

Research is crucial to implement evidence-based health interventions for control of non-communicable diseases (NCDs). This study aims to assess main features of randomized controlled trials (RCTs) for control of NCDs, and to identify gaps in clinical research on NCDs between high-income and less developed countries. The study included 1177 RCTs in 82 Cochrane Systematic reviews (CSRs) and evaluated interventions for adults with hypertension, diabetes, stroke, or heart diseases. Multivariate logistic regression analyses were conducted to explore factors associated with risk of bias in included RCTs. We found that 78.2% of RCTs of interventions for major NCDs recruited patients in high-income countries. The number of RCTs included in the CSRs was increasing over time, and the increasing speed was more noticeable for RCTs conducted in middle-income countries. RCTs conducted in less developed countries tended to be more recently published, less likely to be published in English, with smaller sample sizes, and at a higher risk of bias. In conclusion, there is still a lack of research evidence for control of NCDs in less developed countries. To brace for rising NCDs and avoid waste of scarce research resources, not only more but also higher quality clinical trials are required in low-and-middle-income countries.

Non-communicable diseases (NCDs) are leading causes of mortality, morbidity and disability globally, and the burden of NCDs is rising rapidly in low-and-middle-income countries (LMICs)^1^,^2^. The myth that NCDs affect mainly people in high income countries is consistently dismissed by available evidence. According to the World Health Organization, NCDs caused 38 million of global deaths in 2012, with 74% occurring in LMICs^3^. In addition, NCDs were responsible for more than 40% of premature deaths under age 70 years, and 82% of the premature deaths occurred in LMICs^3^. Therefore, the United Nations held a high-level meeting on NCDs in 2013, and recommended a shift of global priority from infectious to non-infectious diseases^4^.

Research is crucial to develop and implement evidence-based health interventions for the prevention and control of NCDs in LMICs, as in high-income countries^5^,^6^. It is well known that most available evidence is from research conducted in high-income countries^7^,^8^. An analysis of Cochrane reviews found that only a very small proportion of trials of interventions for NCDs were conducted in LMICs^9^. Evidence from research in high-income countries may not be directly applicable to LMICs^10^,^11^. For example, empirical data indicated that effect sizes in clinical trials from more developed countries may be different from less developed countries^12^.

High quality randomized controlled trials (RCTs) provide the most valid evidence for the prevention and control of NCDs^13^. Although previous studies considered the amount and effect sizes of RCTs conducted in LMICs^9^,^12^, RCTs conducted in high-income countries and in LMICs have not been comprehensively compared in terms of sample sizes, publication languages, and risk of bias. The purpose of this study is to assess main features of RCTs for the control of NCDs, and to identify gaps in clinical research on NCDs between high-income and less developed countries.

## Methods

### Eligibility criteria

We included recently updated (since 2010) Cochrane Systematic reviews (CSRs) that evaluated treatment interventions for adult patients with the following chronic conditions: hypertensive disorders, Type 2 diabetes mellitus, stroke, or heart diseases. We exclude CSRs that evaluated interventions exclusively in children, infants or pregnant women. We also excluded CSRs of interventions primarily for the prevention of chronic conditions. There was no restriction on the primary outcome measures and the length of follow up.

### Selection and data extraction

We searched Cochrane Database of Systematic Reviews in Cochrane Library (Issue 4 of 12, 2014) to identify eligible CSRs. The search strategy included a combination terms of “hypertension OR hypertensive OR diabetes OR diabetic OR stroke OR cardiovascular OR cerebrovascular” in Title, Abstract, or Keywords. Using this search strategy, we searched the Cochrane Database and transferred the initial yield into a bibliographic database (Endnotes). One researcher (HF) applied the inclusion and exclusion criteria to identify relevant CSRs, and a second reviewer (FS) was involved when it was difficult to decide the eligibility of a CSR.

Data extraction was conducted by one researcher (HF) and then checked by a second researcher (FS). Discrepancy was addressed by discussion. The following data were obtained from the included CSRs: year as up-to-date, country of the corresponding author of CSRs, language restrictions for study inclusion, and chronic conditions addressed. From RCTs included in the CSRs, we extracted data on types of interventions, year of publication, sample size, country origin, publication language, and results of risk of bias assessment.

Quality of all RCTs included in CSRs was assessed using the Cochrane Collaboration’s tool for assessing risk of bias^13^. Specifically, the Cochrane quality parameters for risk of bias are designed to answer the following six questions. (1) Was the allocation sequence adequately generated? (2) Was allocation adequately concealed? (3) Was knowledge of the allocated intervention adequately prevented during the study? (4) Were incomplete outcome data adequately addressed? (5) Are reports of the study free of suggestion of selective outcome reporting? (6) Was the study apparently free of other problems that could put it at a high risk of bias? For each of these questions, systematic reviewers’ answers may be ‘Yes’, ‘No’ or ‘Unclear’, based on information available from included RCTs. If the answer is ‘Yes’, it indicates a low risk of bias. In this study, we used results of risk of bias assessment for the first five questions, because risk of other biases (the last question) was inconsistently assessed in the included CSRs.

### Data synthesis and analysis

Data extracted from the included CSRs and RCTs were summarized by tabulations. RCTs included in the relevant CSRs were categorized by year and language of publication, country in which they were conducted, and risk of bias. The definition of high-income, middle-income and low-income country was accordance with the World Bank’s country classification^14^. Mixed-effects, multivariate logistic regression analyses were also conducted to explore factors associated with risk of bias of included RCTs^15^.

## Results

**Figure 1:**
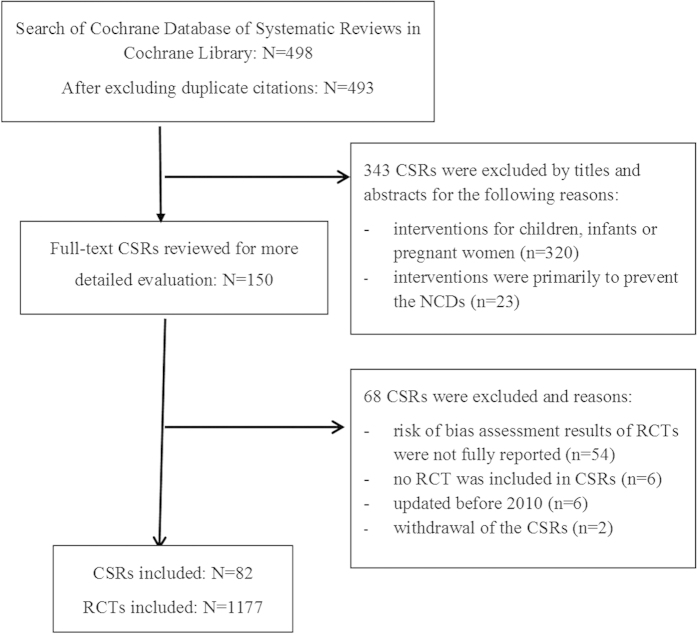
Selection of relevant Cochrane Systematic Reviews (CSRs).

A total of 498 reviews from 8440 records were retrieved on April 2014. We screened 493 reviews after removal of duplicates. Based on their titles and abstracts, a total of 82 reviews met the inclusion criteria and made up the data set^16^,^17^,^18^,^19^,^20^,^21^,^22^,^23^,^24^,^25^,^26^,^27^,^28^,^29^,^30^,^31^,^32^,^33^,^34^,^35^,^36^,^37^,^38^,^39^,^40^,^41^,^42^,^43^,^44^,^45^,^46^,^47^,^48^,^49^,^50^,^51^,^52^,^53^,^54^,^55^,^56^,^57^,^58^,^59^,^60^,^61^,^62^,^63^,^64^,^65^,^66^,^67^,^68^,^69^,^70^,^71^,^72^,^73^,^74^,^75^,^76^,^77^,^78^,^79^,^80^,^81^,^82^,^83^,^84^,^85^,^86^,^87^,^88^,^89^,^90^,^91^,^92^,^93^,^94^,^95^,^96^,^97^. The 82 relevant CSRs included 1177 RCTs. Figure 1 shows the process of the CSRs selection.

The main characteristics of the included CSRs are summarized in Table 1. Corresponding authors of the CSRs were mostly from institutions in high-income countries (75.6%). The institutional affiliations of corresponding authors were in an upper-middle-income country for 19 CSRs and in a lower-middle-income country for only one CSR. Of the 82 CSRs, stroke or related vascular conditions were addressed in 36 (43.9%), hypertension disorders in 17 (20.7%), heart disease in 13 (15.9%), and diabetes in 16 (19.5%). Twelve point two percent of the included CSRs were updated as to 2010, 29.3% up to 2011, 34.1% up to 2012, and 24.4% up to 2013 or 2014. The median number of RCTs included in these CSRs was 9 (interquartile 4 to 17). Language restriction was explicitly not used in 75 (91.5%) CSRs, and it was unclear in six CSRs. Only one CSR explicitly applied language restriction to included trials published in European languages (including English, German, Dutch, French, Italian, Portuguese or Spanish).

### Randomised controlled trials included

The main characteristics of the 1177 RCTs are shown in Table 2. Most of the RCTs (78.2%) were conducted in high-income countries, and only 18.3% of the 1177 RCTs recruited patients in middle-income countries (none from low-income countries). The proportion of RCTs in mixed-income countries (multiple countries belonging to different income groups) was 3.5%. The total number of patients in the 1177 RCTs was 511307; 76.3% were recruited in high-income countries, 19.1% in mixed countries, and only 4.6% in middle-income countries. The sample size of individual RCTs ranged from 4 to 22576 (median 85, interquartile range: 40 to 198). RCTs with a sample size ≥500 accounted for 9.8% of RCTs in high-income countries and only 1.9% in middle-income countries (Table 2).

Of the 1177 RCTs, most were published in English (89.8%), and the proportion of RCTs published in Chinese was 8%, while other languages accounted less than 3%. When comparing the language of publication of RCTs conducted in different countries, except China, English dominated the language of publication in most of the countries. For the 124 RCTs conducted in China, 92 (74%) were published in Chinese language (including one published in both English and Chinese).

**Figure 2:**
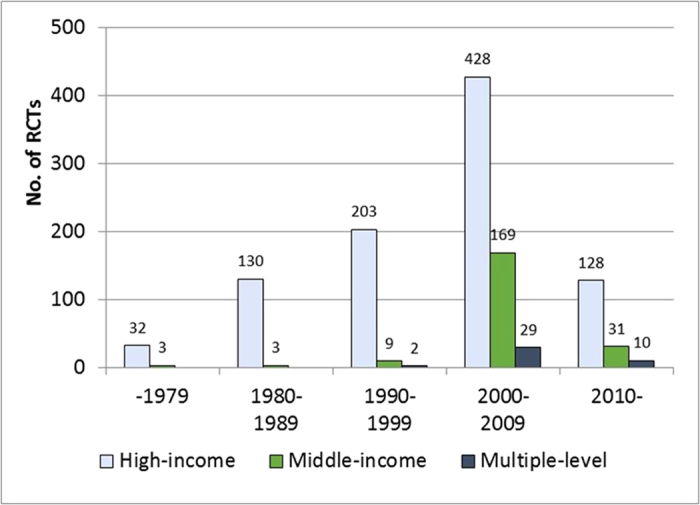
Year of publication of the included RCTs by income levels. Note: Some relevant RCTs published since 2010 may not be included in the CSRs.

The included RCTs were published from 1962 to 2013, although most were published since 2000 (67.5%). The number of RCTs included in the CSRs was increasing over time, and the increasing speed was noticeable for RCTs conducted in middle-income countries and mixed-income countries. The ratio of RCTs conducted in middle-income countries to RCTs conducted in high-income countries was only 4.4% (9:203) for RCTs published from 1990 to1999, and it increased to 36% (200:556) for those published since 2000 (Figure 2). Twenty-nine of the 626 RCTs published from 2000 to 2009 were conducted in multiple income groups of countries, while there were only two such RCTs published from 1990 to 1999 and none before 1990 (Fig. 2).

### Quality of included RCTs

Of the 1177 RCTs, the proportion of RCTs with a low risk of bias was 45.0% in terms of sequence generation, 33.2% regarding allocation concealment, 37.2% regarding blinding, 57.6% regarding incomplete outcome, and 44.2% about reporting bias (Table 3). The validity of the included RCTs tended to improve over time, although the proportion of low risk of bias was higher in RCTs published before 1980 compared with RCTs published from 1980 to 1989.

The proportion of low risk of bias was higher for RCTs conducted in high-income countries than in middle-income countries, and the proportion of low risk of bias was highest in RCTs conducted in mixed-income countries. In addition, RCTs with larger sample sizes tended to have a low risk of bias (Table 3). The proportion of RCTs with low risk of bias was highest in RCTs published in English and lowest in RCTs published in Chinese. In terms of allocation concealment, for example, the frequency of low risk of bias was 36.1% for RCTs published in English, 3.3% for RCTs published in Chinese, and 20.7% for RCTs published in languages other than English or Chinese. Regarding reporting bias, the proportion of low risk of bias was 47.4% for RCTs published in English, 8.7% for RCTs published in Chinese, and 37.9% for RCTs published in other languages (see Table 3).

The results of mixed-effects, multivariate, logistic regression analysis are presented in Table 4. The dependent variable used in the analysis was a dummy variable for high study validity, defined as at least four of the five bias items were judged to be low. The results indicated that the study validity was high (that is, at low risk of bias) in RCTs with larger sample sizes, published in English and more recently. The study validity was relatively low in RCTs conducted in middle-income countries (P = 0.005). After adjusting for other variables, the difference in study validity was statistically non-significant between RCTs conducted in mixed-income countries and those in high-income countries (P = 0.11).

## Discussion

The results of the current study are consistent with findings from previous studies, indicating that clinical research of interventions for NCDs has been conducted mainly in high-income countries, and there is a lack of research evidence from LMICs^7^,^8^,^9^,^11^. We found that 78.2% of RCTs of treatment interventions for major NCDs recruited patients in high-income countries, 18.3% in middle-income countries, 3.5% in mixed-income countries, and none in low-income countries. In the current study, we also systematically examined the main features of individual RCTs by income group, including sample size, year and language of publication, and risk of bias. Compared with RCTs conducted in high-income countries, RCTs conducted in middle-income countries tended to be more recently published, less likely to be published in English, with smaller sample sizes, and at a higher risk of bias.

Although the proportion of research evidence from LMICs is still very low, the number of RCTs on NCDs treatment interventions has been increasing in middle-income countries, which was similar to findings from other studies. A study reported an 18% increase in the proportion of health-related publications with authors in upper-middle-income countries between 2002 and 2011, and an 8% decreased in the proportion of publications with authors in high-income countries during the same period^8^.

It is important to note that the quality of clinical trials in middle-income countries is generally lower compared with clinical trials in high-income countries. This was in line with findings from previous studies^98^. In addition, RCTs conducted in middle-income countries were more likely to be published in languages other than English, and studies published in languages other than English may be less likely to be included in systematic reviews^99^. Scarce resources for research in developing countries would be wasted if findings from many clinical trials there conducted could not be trusted or included in systematic reviews due to poor design and reporting quality^100^,^101^. Therefore, the quality of clinical research in LMICs needs to be appropriately addressed. Not only more but also high quality trials are required in less developed countries.

There was recently an increase in the number of multi-national studies conducted in mixed-income countries. Studies in mixed-income countries tended to have large sample sizes and a low risk of bias. Because collaboration between researchers in more and less developed countries will usually be required to conduct studies in mixed-income countries, such studies should be promoted to overcome problems caused by limited research capacity in less developed countries.

### Generalizability of evidence from high-income countries to LMICs

Currently, high quality research evidence on the effectiveness of interventions for the control of NCDs is mainly from high-income countries. To tackle NCDs cost-effectively in LMICs, policy and practice decisions will often have to be based on research evidence generated from high-income countries^102^. However, evidence from high-income countries may not be generalizable to LMICs^10^.

Empirical evidence indicated the existence of country specific effects of medical interventions. For example, studies conducted in the United States (US) on average reported smaller treatment effects of cardiorenal drugs compared with studies in the non-US countries^103^. A meta-epidemiological study found that RCTs conducted in less developed countries tended to report more favourable results than trials in more developed countries^12^. The genuine difference in treatment effects between countries could not be ruled out in many cases. For example, it has been suggested that some interventions may be no more effective than usual care in high-income countries, but may be more effective than usual care in less developed countries^104^.

### Limitations

RCTs that were not included in the Cochrane reviews could not be considered in this study. It is possible that some trials published in languages other than English may have not been included in the relevant Cochrane systematic reviews. Therefore, the proportion of RCTs conducted in LMICs may have been under-estimated in the current study.

We did not include CSRs that evaluated interventions for primary prevention, or interventions exclusively in children, infants or pregnant women. Therefore, findings from the current study may not be generable to trials of interventions for primary prevention or trials that exclusively included children or pregnant women.

The treatment effects of interventions were not compared in the current study, which was examined in a recent meta-epidemiological study^12^. Given the limited resources and time, we only included CSRs that evaluated treatment interventions for patients with stroke, hypertension, heart disease and diabetes.

## Conclusions

Clinical research of interventions for NCDs has been conducted mainly in high-income countries, and there is a lack of research evidence from LMICs. RCTs conducted in LMICs were less likely to be published in English, with smaller sample sizes, and at a higher or unclear risk of bias than trials in high-income countries. To brace for rising NCDs and avoid waste of scarce research resources, not only more but also higher quality clinical trials are required in LMICs.

## Additional Information

**How to cite this article**: Fan, H. and Song, F. An assessment of randomized controlled trials (RCTs) for non-communicable diseases (NCDs): more and higher quality research is required in less developed countries. *Sci. Rep.* **5**, 13221; doi: 10.1038/srep13221 (2015).

## Figures and Tables

**Table 1:** The characteristics of the included Cochrane Systematic Reviews (CSRs).

Total	Number of CSRs (%)
	82
Country of CSR corresponding authors	
High-income countries	62 (75.6%)
Middle-income countries	20 (24.4%)
Chronic conditions addressed:	
Stroke/vascular disorders	36 (43.9%)
Hypertensive disorders	17 (20.7%)
Heart disease	13 (15.9%)
Diabetes	16 (19.5%)
Year of updated to:	
2010	10 (12.2%)
2011	24 (29.3%)
2012	28 (34.1%)
2013–2014	20 (24.4%)
Number of RCTs included:	
1–4	26 (31.7%)
5–9	16 (19.5%)
10–14	15 (18.3%)
15–19	9 (11.0%)
≥20	16 (19.5%)
Any language restriction:	
No	75 (91.5%)
Yes	1 (1.2%)
Unclear	6 (7.3%)

**Table 2:** The characteristics of randomised controlled trials included in the CSRs by income group of countries.

	High-income countries	Middle-income	Multiple countries	Total
Total RCTs	921	215	41	1177
Number of total patients	390362	23279	97666	511307
Chronic conditions:				
Stroke	317	93	7	417
Heart disease	280	86	6	372
Hypertension	168	6	2	176
Diabetes	156	30	26	212
Intervention type:				
Pharmacological	403	121	37	561
Rehabilitation	226	39	3	268
Disease management	134	17	1	152
Other	158	38	0	196
Published before 2000	365	15	2	382
Published since 2000	556	200	39	795
Published in English	895	121	41	1057
Published in other languages	26	94	0	120
CSR corresponding authors:				
High-income countries	824	176	32	1032
Middle-income countries	97	39	9	145
RCTs with sample size <500	839	211	12	1062
RCTs with sample size ≥500	82	4	29	115

**Table 3:** The number and proportion of RCTs with low risk of bias by income, publication language, sample size and year of publication.

Total (n = 1177)	Sequence generation	Allocation concealment	Blinding	Incomplete outcome	Reporting
	530 (45.0%)	391 (33.2%)	438 (37.2%)	678 (57.6%)	520 (44.2%)
Country income levels					
High-income (n = 921)	446 (48.4%)	323 (35.1%)	344 (37.4%)	536 (58.2%)	431 (46.8%)
Middle-income (n = 215)	58 (27.0%)	40 (18.6%)	69 (32.1%)	117 (54.4%)	58 (27.0%)
Multiple income (n = 41)	26 (63.4%)	28 (68.3%)	25 (61.0%)	25 (61.0%)	31 (75.6%)
Publication language of RCTs					
English (n = 1056)	506 (47.9%)	382 (36.1%)	415 (39.3%)	619 (58.6%)	501 (47.4%)
Chinese (n = 92)	14 (15.4%)	3 (3.3%)	15 (16.5%)	43 (47.3%)	8 (8.7%)
Other languages (n = 29)	10 (34.5%)	6 (20.7%)	8 (27.6%)	16 (55.2%)	11 (37.9%)
Total sample size of RCTs					
<50 (n = 366)	128 (35.0%)	95 (26.0%)	126 (34.4%)	213 (58.2%)	141 (38.5%)
50–99 (n = 295)	127 (43.1%)	75 (25.4%)	102 (34.6%)	163 (53.9%)	115 (39.0%)
100–199 (n = 224)	103 (46.0%)	75 (33.5%)	84 (37.5%)	136 (60.7%)	101 (45.1%)
200–499 (n = 177)	87 (49.2%)	66 (37.3%)	68 (38.4%)	104 (58.8%)	90 (50.9%)
≥500 (n = 115)	85 (73.9%)	80 (69.6%)	58 (50.4%)	62 (53.9%)	73 (63.5%)
Year of publication of RCTs					
Before 1980 (n = 35)	11 (31.4%)	12 (34.3%)	13 (37.1%)	19 (54.3%)	17 (48.6%)
1980–1989 (n = 133)	39 (29.3%)	24 (18.1%)	40 (30.1%)	50 (37.6%)	40 (30.1%)
1990–1999 (n = 214)	81 (37.9%)	51 (23.8%)	75 (35.1%)	103 (48.1%)	94 (43.9%)
2000–2009 (n = 626)	301 (48.1%)	231 (36.9%)	243 (38.8%)	402 (64.2%)	281 (44.9%)
Since 2010 (n = 169)	98 (58.0%)	73 (43.2%)	67 (39.6%)	104 (61.5%)	88 (52.1%)

**Table 4:** Results of mixed-effects, multivariate, logistic regression analysis: association between study validity and country income, sample size, year and language of publication.

	Odds ratio (95% CI)	P-value
Middle-income country (1 for middle-income country; 0 for high-income country)	0.444 (0.251, 0.786)	0.005
Multiple-income countries (1 for mixed-income; 0 for high-income country)	1.987 (0.856, 4.609)	0.110
Sample size (1 for sample size ≥200; 0 for sample size <200)	2.643 (1.751, 3.988)	<0.001
Year of publication (1 for published since 2005; 0 for published before 2005)	3.173 (2.123, 4.743)	<0.001
Published in English (1 for English; 0 for non-English)	5.532 (1.892, 16.178)	0.002

Note: Dependent variable is defined as at least 4 of the 5 quality items being low risk of bias.
